# Ferroptosis in NAFLD: insights and the therapeutic potential of exercise

**DOI:** 10.3389/fmed.2025.1462145

**Published:** 2025-03-26

**Authors:** Chang Li, Dongkun Deng, Qingfeng Jiang, Jiaming Shi, Lin Xu, Yufei Liu

**Affiliations:** ^1^Graduate School, Harbin Sport University, Harbin, Heilongjiang, China; ^2^College of Human Sport Science, Harbin Sport University, Harbin, Heilongjiang, China

**Keywords:** ferroptosis, non-alcoholic fatty liver disease, cell death, exercise, mechanism

## Abstract

Ferroptosis, a distinct form of non-apoptotic cell death driven by iron accumulation, has garnered significant attention in recent years. Emerging evidence suggests that ferroptosis in hepatocytes may serve as a pivotal trigger in the pathogenesis of non-alcoholic fatty liver disease (NAFLD). Importantly, inhibiting ferroptosis has shown promising potential in slowing the progression of NAFLD. Concurrently, exercise, a cornerstone in the prevention and management of chronic diseases, plays a critical role in regulating disease progression. As such, the modulation of ferroptosis through exercise represents a promising avenue for developing innovative therapeutic strategies. This review aims to systematically elucidate the conceptual framework and molecular mechanisms underlying ferroptosis, with particular emphasis on its pathophysiological role in NAFLD. We have systematically summarized the effects of exercise on ferroptosis regulation through multiple molecular mechanisms, including upregulation of antioxidant defense systems via activation of NRF2, GPX4, and SLC7A11 signaling pathways; and modulation of iron metabolism through FPN-mediated iron homeostasis regulation. These findings not only provide valuable insights into the molecular basis of exercise-induced protection against ferroptosis-mediated cellular damage but also offer novel perspectives for future investigations into exercise-based interventions for NAFLD management. This work thereby contributes to the advancement of therapeutic strategies in the field of metabolic liver diseases.

## 1 Introduction

Non-alcoholic fatty liver disease (NAFLD) has become one of the most prevalent and serious chronic liver diseases worldwide, and represents a leading cause of liver disease globally, significantly impairing patients’ quality of life and, in severe cases, leading to life-threatening complications such as cirrhosis and hepatocellular carcinoma. According to research employing Markov models to forecast the epidemiology of diseases over the coming decades, the burden of advanced liver diseases (1), such as cirrhosis and hepatocellular carcinoma, caused by NAFLD is projected to more than double globally from 2016 to 2030, reflecting an exponential increase in the disease burden. The ongoing global rise in obesity and type 2 diabetes rates is a major driver of the increasing incidence of NAFLD, thereby imposing a substantial economic and healthcare burden on individuals and society, highlighting the urgent need for effective interventions. Effective management of NAFLD requires a deeper understanding of its pathogenesis and the development of mechanism-based therapeutic strategies. Currently, ferroptosis, a recently discovered form of regulated cell death, has gained significant attention in diverse fields such as life sciences, medicine, and chemistry, and has been implicated in the pathogenesis of various major diseases, including cancers, neurodegenerative disorders, and metabolic diseases (2–5).

## 2 Ferroptosis

In 2012, Dixon et al. first described ferroptosis (6), a novel form of regulated cell death characterized by its distinct mechanisms and morphological features, setting it apart from necrosis, apoptosis, and autophagy. The morphological hallmarks of ferroptosis are predominantly observed in mitochondria, which display characteristic changes such as shrinkage, increased membrane density, and a marked reduction or complete loss of mitochondrial cristae. The biological hallmarks of ferroptosis include the depletion of glutathione (GSH) and the subsequent reduction in the activity of glutathione peroxidase 4 (GPX4), a key enzyme responsible for lipid peroxide detoxification. In the absence of GSH, the activity of enzymes such as NADPH oxidase (NOX) may be upregulated. NOX is a key enzymatic source of reactive oxygen species (ROS) within cells (7), and its increased activity leads to elevated ROS levels, which subsequently trigger ferroptosis. A comparison of the key features of different forms of programmed cell death, including ferroptosis, apoptosis, necrosis, and autophagy, is summarized in Table 1.

**TABLE 1:** Comparison of programmed cell death modes.

Programmed cell death pathways	Morphological features	Biochemical characteristics
Apoptosis	Cellular shrinkage, preserved plasma membrane integrity, nuclear condensation, fragmentation of nuclear DNA	Members of the BCL-2 family, activation of caspases, ROS production
Necroptosis	Cellular swelling, rupture of the plasma membrane, lysis of the nucleus	Activation of RIPK1, RIPK3, and MLKL;ROS production, Release of DAMP
Ferroptosis	Mitochondrial atrophy, increased mitochondrial membrane density, and reduced or absent mitochondrial cristae	GSH depletion, GPX4 activity reduction, ROS accumulation, lipid peroxidation
Necrosis	Nuclear swelling, dissolution of the nuclear membrane and cytoplasmic granule membrane, and rupture of the cell membrane	HSPs release, ATP release, inflammasome activation, pro-inflammatory factor release
Pyroptosis	Cell membrane rupture, cell swelling, intact nucleus, nuclear DNA fragmentation	Caspase, caspase-3, caspase-1 activation Pro-inflammatory factors IL-1β and IL-18 release
Autophagy	Cell membrane intact, cytoplasmic vacuolization, chromatin not aggregated	The conversion from LC3-I to LC3-II and the increase in autophagic flux.

BCL-2, B-cell lymphoma-2; caspase, cysteine-aspartic proteases; ROS, reactive oxygen species; RIPK1, receptor-interacting serine/threonine-protein kinase 1; RIPK3, receptor-interacting serine/threonine-protein kinase 3; MLKL, mixed lineage kinase domain-like pseudokinase; DAMPs, damage-associated molecular patterns; GSH, glutathione; GPX4, glutathione peroxidase 4; HSPs, heat shock proteins; IL-1β, interleukin-1 beta; IL-18, interleukin-18; LC3-I/LC3-II, microtubule-associated proteins 1A/1B light chain 3 (autophagy markers).

### 2.1 The sequential mechanism of ferroptosis

#### 2.1.1 Iron metabolism

**FIGURE 1:**
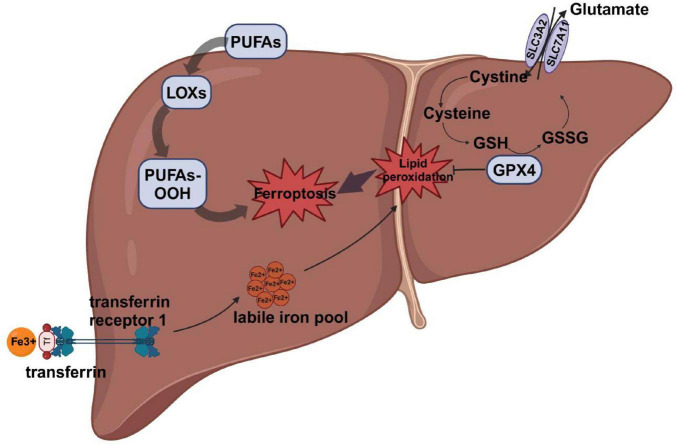
The main mechanism of ferroptosis. Iron Uptake and Metabolism:Fe^3 +^ binds to TF and is transported into the cell via TFR1. Fe^3 +^ is released from TF and reduced to Fe^2 +^ by lysosomal reductases. Fe^2 +^ enters the LIP, where it catalyzes ROS production via the Fenton reaction. Lipid Peroxidation: PUFAs are incorporated into membrane phospholipids. LOXs or Fe^2 +^-catalyzed ROS oxidize PUFAs to form PUFA-PLOOH, driving membrane damage. System Xc?/GSH/GPX4 Axis:System Xc? imports cystine in exchange for glutamate, supporting GSH synthesis. GSH is required for GPX4 activity, which reduces PLOOH to non-toxic lipid alcohols, preventing ferroptosis. Inhibition of System Xc? depletes GSH, inactivates GPX4, and leads to lipid peroxidation and ferroptosis. TF, transferrin;TFR1, transferrin receptor 1; LIP, labile iron pool; PUFAs, polyunsaturated fatty acids; LOXs, lipoxygenases; GSH, glutathione; GPX4, glutathione peroxidase 4; ROS, reactive oxygen species; SLC3A2, solute carrier family 3 member 2; SLC7A11, solute carrier family 7 member 11;GSSG, glutathione disulfide.

Iron is a vital trace element essential for numerous biological processes, including oxygen transport, energy production, DNA synthesis, and cellular respiration (8). Disruptions in iron metabolism can impair normal physiological functions and are implicated in the pathogenesis of various diseases, including anemia, neurodegenerative disorders, and cancer. Iron metabolism is tightly regulated by a sophisticated control system that maintains iron homeostasis through precise regulation of iron absorption, storage, and excretion (9). Under conditions of iron overload, iron regulatory proteins (IRPs) play a central role in modulating the expression of genes involved in iron import, storage, and export, thereby maintaining cellular iron homeostasis (10). Under physiological conditions, transferrin (TRF) binds and transports iron primarily in its ferric form (Fe^3 +^) to various tissues and cells. Transferrin-bound iron (TBI) is internalized via binding to transferrin receptor 1 (TFR1) on the cell surface. Following internalization, Fe^3 +^ is reduced to Fe^2 +^ by six-transmembrane epithelial antigen of the prostate 3 (STEAP3) and subsequently transported into the cytoplasm by divalent metal transporter 1 (DMT1). The released iron can be utilized for cellular processes, stored in the labile iron pool (LIP), or exported to the extracellular space via ferroportin (FPN), the only known mammalian iron exporter (10). However, persistently elevated Fe^2 +^ levels can trigger the Fenton reaction, in which Fe^2 +^ reacts with hydrogen peroxide to generate highly reactive hydroxyl radicals, leading to the accumulation of reactive oxygen species (ROS) and enhanced lipid peroxidation (11). Additionally, iron is a cofactor for lipoxygenases (LOXs), enzymes that catalyze the oxidation of polyunsaturated fatty acids, leading to the production of lipid ROS, such as lipid hydroperoxides. The resulting lipid peroxidation causes extensive damage to cellular and mitochondrial membranes, ultimately triggering ferroptosis (12) (Figure 1).

#### 2.1.2 Lipid peroxidation

Lipid peroxidation, a process driven by the oxidation of polyunsaturated fatty acids (PUFAs) in cellular membranes, is the primary driver of ferroptosis. Lipid hydroperoxides, generated primarily through the activity of lipoxygenases (LOXs), are key mediators of cellular dysfunction and death (13). Thus, LOXs are critically involved in the induction of ferroptosis by catalyzing the production of lipid hydroperoxides. Pharmacological inhibition of LOXs has been demonstrated to attenuate ferroptosis, highlighting the enzyme’s central role in this cell death pathway (14). Genetic knockout of 12/15-LOX or pharmacological inhibition with baicalein has been shown to protect mice from traumatic brain injury and improve neurological outcomes, further supporting the role of LOXs in ferroptosis (15, 16). These findings underscore the significant role of LOXs in ferroptosis. The extent of ferroptosis is directly influenced by the degree of lipid peroxidation, which is largely determined by the abundance and oxidation susceptibility of polyunsaturated fatty acids (PUFAs) in cellular membranes. Ferroptosis is driven by the peroxidation of specific phospholipid membranes, a process that requires the incorporation of PUFAs into membrane phospholipids. This incorporation renders membranes susceptible to lipid peroxidation through enzymatic (e.g., LOXs) and non-enzymatic (e.g., Fenton reaction) mechanisms, ultimately leading to cell death via the accumulation of toxic peroxidation products (17). Pre-treatment of cells with deuterated PUFAs (D-PUFAs), which are resistant to oxidation due to the kinetic isotope effect, effectively prevents lipid peroxidation and inhibits ferroptosis (18). Future research may explore therapeutic strategies targeting PUFA metabolism, such as inhibiting their incorporation into phospholipid membranes or developing compounds that block their oxidation, as potential approaches to prevent ferroptosis.

#### 2.1.3 Antioxidant system

##### 2.1.3.1 System Xc-/GSH/GPX4 axis

**FIGURE 2:**
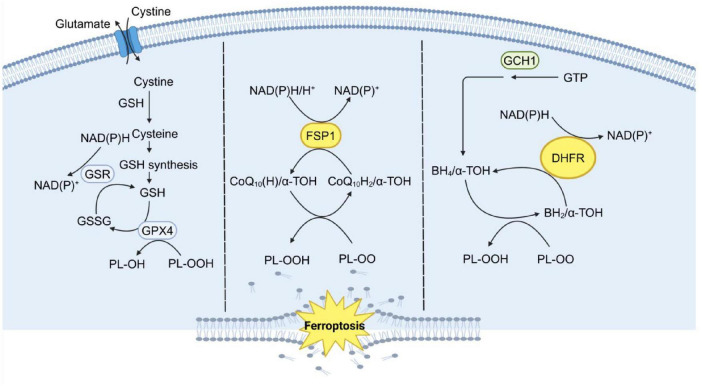
The three major systems that control ferroptosis. Ferroptosis is regulated by three major antioxidant axes: the System Xc-/GSH/GPX4 axis, the GCH1/BH4/DHFR axis, and the FSP1/CoQ10/NADH axis, all of which are driven by NADPH. System Xc-/GSH/GPX4 Axis: This axis relies on the cystine-glutamate antiporter system Xc-, which imports cystine for GSH synthesis. GSH is a critical substrate for GPX4, an enzyme that reduces PLOOH to non-toxic lipid alcohols, thereby preventing lipid peroxidation and ferroptosis. GCH1/BH4/DHFR Axis: This axis involves GCH1, which catalyzes the production of BH4, a potent antioxidant. BH4 helps regenerate reduced forms of cofactors and scavenges ROS. DHFR further supports this pathway by maintaining BH4 levels, thus enhancing cellular antioxidant capacity. FSP1/CoQ10/NADH Axis: FSP1 utilizes NADH to reduce CoQ10 to CoQ10H2, a potent lipophilic antioxidant. CoQ10H2 directly neutralizes lipid PLOO and inhibits lipid peroxidation, independent of the GPX4 pathway. This axis is supported by the mevalonate pathway, which generates CoQ10. These three parallel metabolic pathways work synergistically to suppress phospholipid peroxidation, a hallmark of ferroptosis, by maintaining redox homeostasis and protecting cellular membranes from oxidative damage. GSH, glutathione; GSR, glutathione reductase; GSSG, glutathione disulfide; GPX4,glutathione peroxidase 4; FSP1, ferroptosis-suppressor protein 1; CoQ10, ubiquinone; CoQ10H2, ubiquinol; α-TOH, α-tocopherol; GTP, guanosine triphosphate; GCH1, guanosine triphosphate cyclohydrolase 1; BH2, dihydrobiopterin; BH4, tetrahydrobiopterin; DHFR, dihydrofolate reductase; PLOO, peroxyl radical; PLOOH, phospholipid hydroperoxides.

The cystine/glutamate antiporter (System Xc-), glutathione (GSH), and glutathione peroxidase 4 (GPX4) form a critical antioxidant axis that plays a central role in regulating ferroptosis. System Xc- is a heterodimeric complex composed of the light chain SLC7A11 and the heavy chain SLC3A2, which mediates the 1:1 exchange of extracellular cystine for intracellular glutamate (19). Following uptake, cystine is reduced to cysteine by thioredoxin reductase 1 (TrxR1) (20), a critical step for glutathione synthesis. Dysregulation of System Xc- impairs cystine uptake, leading to cysteine and GSH depletion, and consequently increasing cellular susceptibility to ferroptosis. GSH, a key cellular antioxidant, serves as an essential cofactor for GPX4 activity, enabling the enzyme to neutralize lipid hydroperoxides. GPX4 specifically reduces phospholipid hydroperoxides, protecting cell membranes from oxidative damage and playing a pivotal role in regulating ferroptosis. By converting lipid hydroperoxides into non-toxic alcohols, GPX4 mitigates lipid peroxidation toxicity and maintains the integrity of the lipid bilayer (Figure 2). Aegul et al. demonstrated that genetic ablation of GPX4 in mice leads to acute renal failure and renal tubular ferroptosis, highlighting the enzyme’s critical role in preventing ferroptosis (21). Erastin, a ferroptosis inducer, depletes GSH levels, thereby reducing GPX4 activity, increasing ROS accumulation, and triggering ferroptosis (22). Erastin induces ferroptosis by inhibiting System Xc-. In contrast, Erastin2 not only selectively inhibits System Xc- but also independently suppresses mTOR and activates the GCN2/ATF4 pathway, enhancing its ferroptosis-inducing effects (23). Thus, Erastin2 exhibits superior potency in inducing ferroptosis through dual targeting of System Xc- and mTOR/GCN2 pathways; however, its efficacy is context-dependent and may vary across cell types and experimental settings. RSL3, another ferroptosis inducer, directly inhibits GPX4 activity, leading to ferroptosis in colorectal cancer cells (24). Targeting ferroptosis as a therapeutic strategy has spurred the development of numerous pharmacological inducers and inhibitors, which are being actively investigated for their potential in treating various diseases (Table 2).

**TABLE 2:** A summary of some inducers and inhibitors of ferroptosis.

Compound	Mechanism of action	References
**Inducer**		
Erastin	System xc−inhibitor	(21, 26)
Erastin2	System xc−inhibitor	(22)
BSO	γGCS inhibitor, depletion of GSH	(27)
RSL3	GPX4 inhibitor	(23)
**Inhibitor**		
Fer-1	Catalytic RTA, prevention of lipid peroxidation	(36–39)
Tβ4	Alleviate oxidative stress, enhance GPX4 activity	(40–42)
GB	Prevent lipid peroxidation, activate Nrf2	(43, 44)
Lip-1	Catalytic RTA, prevention of lipid peroxidation	(37, 45–47)
α-tocotrienol	Prevent lipid peroxidation, enhance antioxidant capacity	(48–51)

BSO, buthionine sulfoximine; RSL3, RAS-selective lethal 3; Fer-1, ferrostatin-1; Tβ4, thymosin β4; GB, ginkgolide B; Lip-1, liproxstatin-1; γGCS, γ-glutamylcysteine synthetase; GSH, glutathione;GPX4, glutathione peroxidase 4; Nrf2, nuclear factor erythroid 2-related factor 2; RTA, radical trapping antioxidant.

##### 2.1.3.2 The GCH1/BH4/DHFR axis

Guanosine triphosphate (GTP) cyclohydrolase 1 (GCH1) catalyzes the initial step in the biosynthesis of tetrahydrobiopterin (BH4), a process that also involves 6-pyruvyl tetrahydrobiopterin synthase (PTPS) and sepiapterin reductase (SPR). BH4 is regenerated from its oxidized form by dihydrofolate reductase (DHFR), ensuring its availability as a cofactor. GCH1, the rate-limiting enzyme in BH4 biosynthesis, catalyzes the first and key regulatory step of this pathway (25). As an essential cofactor, BH4 plays a critical role in numerous physiological and pathological processes, including the modulation of oxidative stress and inflammatory responses (26). Treatment with the ferroptosis inducer Erastin led to a significant reduction in BH4 levels, whereas RSL4 treatment did not induce any notable changes in BH4 levels. Elevated GCH1 mRNA expression was observed in colorectal cancer, suggesting a potential link between GCH1 activity and oxidative stress-induced cell death. Erastin treatment suppressed GCH1 expression, correlating with the observed reduction in BH4 levels. The GCH1/BH4 pathway is implicated in Erastin-induced ferroptosis, with its deficiency exacerbating lipid peroxidation in colorectal cancer cells (27). Furthermore, following GPX4 ablation, BH4 levels were elevated, and GCH1 overexpression resulted in a modest increase in free thiols and glutathione levels, even in the presence of the glutathione synthesis inhibitor L-Buthionine-sulfoximine (L-BSO). These findings align with data from GPX4 ablation studies, including those involving BH4 supplementation. Thus, the GCH1/BH4 pathway represents an endogenous antioxidant mechanism that operates independently of the GPX4/GSH axis and plays a critical role in modulating ferroptosis (28).

##### 2.1.3.3 FSP1 CoQ10/NADH axis

Ferroptosis-suppressor protein 1 (FSP1), coenzyme Q10 (CoQ10), and nicotinamide adenine dinucleotide (NADH) constitute a parallel antioxidant system that functions independently of GPX4 to suppress ferroptosis. FSP1, a key resistance factor against ferroptosis, functions as a plasma membrane-associated redox enzyme that utilizes NADH to reduce CoQ10, thereby generating antioxidants and inhibiting lipid peroxidation. Loss of FSP1 does not affect System Xc- or glutathione synthesis but, even in the presence of functional GPX4, results in elevated phospholipid oxidation (29). Coenzyme Q10 (CoQ10), a lipophilic molecule, is predominantly localized in the inner mitochondrial membrane, where it plays a critical role in electron transport and antioxidant defense. The reduced form of CoQ10 (CoQ10H2) acts as a potent antioxidant by scavenging free radicals, thereby preventing lipid peroxidation. Additionally, CoQ10H2 can indirectly promote the generation of α-tocotrienol, which further inhibits ferroptosis through its radical-scavenging activity (30). CoQ2, a key enzyme in the CoQ10 biosynthesis pathway, catalyzes the initial step in the synthesis of coenzyme Q10. Genetic knockout or pharmacological inhibition of CoQ2, even in the context of FSP1 overexpression, fails to prevent ferroptosis. In contrast, soluble analogs of coenzyme Q10 effectively inhibit ferroptosis and lipid peroxidation, highlighting the therapeutic potential of targeting this pathway (29, 31). These findings demonstrate that the FSP1/CoQ10 axis functions as a parallel antioxidant system to GPX4, playing a critical role in regulating ferroptosis.

##### 2.1.3.4 Other molecular mechanisms

In addition to GPX4, several regulatory pathways, including the PERK-NRF2-HO-1, AMPK/GSK-3β/NRF2, and HSF1-HSPB1 axes, play critical roles in modulating ferroptosis independently of the GPX4 system. In colorectal cancer, the ER stress inhibitor 4-PBA effectively suppresses erastin-induced ferroptosis and downregulates the expression of nuclear factor E2-related factor 2 (NRF2) and heme oxygenase-1 (HO-1). NRF2 is directly phosphorylated by protein kinase R-like ER kinase (PERK), a key mediator of the NRF2-HO-1 signaling pathway activation in response to erastin. Erastin triggers ferroptosis in colorectal cancer cells by activating the PERK-NRF2-HO-1 signaling pathway, thereby exerting potent anti-tumor effects (32). Under conditions of glucose deprivation, AMPK is activated, initiating a protective energy stress response that mitigates ferroptosis by impairing the synthesis of polyunsaturated fatty acids (PUFAs), essential drivers of lipid peroxidation (33). This mechanism has been demonstrated in the context of myocardial ischemia-reperfusion injury (MIRI), where AMPK activation plays a protective role against ferroptosis. Dexmedetomidine protects against MIRI-induced ferroptosis by activating NRF2 through the AMPK/GSK-3β signaling pathway, which involves the phosphorylation of AMP-activated protein kinase (AMPK) and subsequent NRF2 activation (34). Heat shock protein β-1 (HSPβ-1) functions as a negative regulator of ferroptosis in iron-dependent cancer cells by mitigating iron-mediated oxidative stress. Knockdown of HSF1 and HSPβ-1 sensitizes cells to Erastin-induced ferroptosis, whereas protein kinase C-mediated phosphorylation of HSPβ-1 exerts a protective effect by reducing iron-dependent ROS accumulation, thereby inhibiting ferroptosis (35).

## 3 Ferroptosis and NAFLD

### 3.1 Ferroptosis and inflammation

Globally, the prevalence of NAFLD continues to rise, encompassing a spectrum of conditions from simple steatosis to NASH, fibrosis, and cirrhosis (40). To investigate therapeutic strategies for NAFLD, researchers have established a high-fat diet-induced rat model, which recapitulates key features of the disease, including lipid accumulation and inflammation. Administration of Tβ4 has been shown to significantly improve lipid metabolism and reduce pro-inflammatory markers, such as TNF-α and IL-6, in high-fat diet-fed rats. This protective effect is mediated through the upregulation of GPX4, a key regulator of ferroptosis that mitigates lipid peroxidation and cellular damage. In contrast, the ferroptosis inducer Erastin exacerbates liver inflammation, while the iron chelator Ferrostatin-1 (Fer-1) attenuates inflammatory responses. Silencing GPX4 expression using small interfering RNA (siRNA) not only induced ferroptosis but also altered the expression of apoptosis-related factors, leading to exacerbated liver injury (45). In a separate study (52), a high-fat diet-induced mouse model was employed to evaluate the effects of ferroptosis inhibitors on NAFLD progression. These inhibitors suppressed key ferroptosis markers, such as ACSL4 and ALOX15, improving lipid metabolism, insulin sensitivity, and reducing liver inflammation. Notably, the iron chelator deferoxamine demonstrated pronounced anti-inflammatory and anti-ferroptotic effects, significantly mitigating liver injury. Bone morphogenetic protein 4 (BMP4), traditionally recognized for its role in bone and cartilage development (53), has recently been implicated in the pathogenesis of NAFLD. Elevated serum BMP4 levels have been observed in patients with obesity and metabolic syndrome, correlating closely with visceral adipose tissue accumulation (54). In both NAFLD mouse models and FFA-induced hepatocyte models, BMP4 upregulation enhances GPX4 expression at both the gene and protein levels, suggesting a protective role against ferroptosis. These findings indicate that BMP4 attenuates hepatic steatosis and inflammation by modulating ferroptosis pathways. *In vitro*, BMP2 overexpression reduced oxidative stress markers, including ROS and malondialdehyde (MDA), alleviating ferroptosis and attenuating liver inflammation (36). Enoyl coenzyme A hydratase 1 (ECH1), a key enzyme in mitochondrial β-oxidation, plays a critical role in mitigating inflammation and oxidative stress. ECH1 overexpression reduces inflammatory and oxidative stress markers, while its silencing diminishes these protective effects. Notably, Ferrostatin-1 (Fer-1) treatment significantly ameliorated NASH symptoms in ECH1 knockdown mice, highlighting the role of ferroptosis in disease progression (43). These findings suggest that ECH1 attenuates inflammation and NASH progression by suppressing ferroptosis (43). In summary, ferroptosis is intricately linked to liver inflammation, and targeting ferroptosis pathways holds promise for reducing inflammation and slowing NAFLD progression.

### 3.2 Ferroptosis and lipid metabolism

Ferroptosis, a recently discovered form of regulated cell death characterized by iron-dependent lipid peroxidation, has emerged as a major focus in biological research due to its implications in various diseases. Emerging evidence highlights the critical role of ferroptosis in modulating lipid metabolism, particularly in the context of metabolic disorders such as NAFLD (45). To elucidate the relationship between ferroptosis and lipid metabolism, researchers established both *in vivo* (mouse) and *in vitro* (hepatocyte) models, enabling comprehensive analysis of ferroptosis-related mechanisms (55). Analysis of ferroptosis-related protein expression revealed that ginkgolide B (GB) treatment significantly upregulated NRF2, a key regulator of ferroptosis, in both liver tissue and hepatocyte models. These findings suggest that NRF2 activation exerts protective effects against ferroptosis by reducing lipid accumulation and oxidative stress, thereby mitigating NAFLD progression. This provides direct evidence linking ferroptosis to the regulation of lipid metabolism. GPX4, a key enzyme in ferroptosis regulation, not only neutralizes polyunsaturated fatty acid hydroperoxides but also mitigates oxidized cholesterol and its esters, highlighting its dual role in ferroptosis and hepatic steatosis (22). Overexpression of GPX4 counteracts the detrimental effects of TRIM59 in NAFLD, while TRIM59 inhibition has been shown to alleviate hepatic steatosis, further underscoring the interplay between these pathways. *In vitro* studies have confirmed that GPX4 overexpression reduces lipid accumulation in hepatocytes, supporting its protective role against steatosis. These findings highlight GPX4’s pivotal role in modulating both ferroptosis and hepatic steatosis. TRIM59 promotes hepatic steatosis and ferroptosis by enhancing GPX4 ubiquitination, leading to its degradation and subsequent loss of antioxidant activity (56). In NAFLD models, upregulation of FMO1 and ferroptosis markers contributes to hepatic lipid accumulation, disrupting lipid metabolism and accelerating disease progression (57). These findings provide deeper insights into the intricate interplay between ferroptosis and lipid metabolism in NAFLD pathogenesis. To explore therapeutic strategies targeting ferroptosis, researchers utilized iron inhibitors and demonstrated that Liproxstatin-1 (Lip-1) significantly reduces hepatic lipid deposition, highlighting its potential for improving lipid metabolism (52). This discovery opens new avenues for developing therapeutic strategies targeting ferroptosis in metabolic diseases such as NAFLD. Fatty acid metabolism, a central process in NAFLD development and progression, has emerged as a key area of research due to its role in lipid homeostasis and disease pathogenesis. Excessive fatty acids overwhelm mitochondrial β-oxidation capacity, leading to mitochondrial dysfunction and the accumulation of toxic lipid intermediates. Unmetabolized fatty acids accumulate as lipid droplets or lipotoxic lipids, inducing endoplasmic reticulum (ER) stress and further exacerbating cellular dysfunction. ER stress triggers the generation of ROS, which exacerbate lipid peroxidation and drive ferroptosis (58). This highlights the intricate connection between ferroptosis and dysregulated fatty acid metabolism in NAFLD pathogenesis. In summary, ferroptosis is a key regulator of lipid metabolism, with its dysregulation contributing significantly to NAFLD progression. Further exploration of ferroptosis mechanisms and pathways may yield novel therapeutic strategies for lipid metabolism-related diseases, including NAFLD.

## 4 Potential effective therapies targeting ferroptosis for NAFLD

### 4.1 Ferroptosis inhibitors

Fer-1 is a selective and potent inhibitor of ferroptosis, known for its ability to scavenge lipid radicals and prevent lipid peroxidation. By reducing lipid hydroperoxides in the presence of ferrous iron, Fer-1 prevents lipid membrane damage and exerts potent anti-ferroptotic effects (37). In a methionine-choline-deficient (MCD) diet-induced NASH mouse model, Fer-1 treatment effectively inhibits ferroptosis by neutralizing lipid ROS, leading to significant reductions in inflammation, fibrosis, and liver injury (38). Fer-1 also reverses hepatotoxicity associated with lipid metabolism disorders by restoring GPX4 activity and inhibiting lipid hydroperoxide accumulation, thereby preventing NAFLD progression (39). Recent studies have demonstrated that Fer-1 treatment not only suppresses lipid ROS accumulation but also protects against mitochondrial dysfunction, further highlighting its therapeutic potential (59).

Tβ4, a G-actin sequestering peptide, plays a pivotal role in regulating actin polymerization and is implicated in a wide range of critical biological processes, including cell migration, wound healing, and inflammation (41). Previous studies have demonstrated that Tβ4 exerts anti-apoptotic and antioxidant effects by inhibiting ROS production and enhancing cellular survival pathways (42). Tβ4 levels are inversely correlated with inflammation and fibrosis in patients with chronic hepatitis B and NAFLD, suggesting a protective role in liver disease progression (44). These findings suggest that Tβ4 may attenuate liver inflammation and fibrosis by suppressing ROS generation and alleviating oxidative stress, thereby protecting against liver injury. Recent studies have confirmed this hypothesis, showing that Tβ4 treatment reduces inflammation, enhances antioxidant defenses, and upregulates GPX4 expression in high-fat diet (HFD)-induced NAFLD rats. These effects collectively inhibit ferroptosis and improve liver function (45).

GB, a terpenoid compound derived from Ginkgo biloba leaves and root bark, is one of the key bioactive constituents known for its therapeutic properties (60). GB exhibits potent anti-inflammatory, antioxidant, and free radical scavenging activities, making it a promising candidate for mitigating oxidative stress-related diseases (46). Recent studies have demonstrated that GB significantly modulates oxidative stress and ferroptosis markers, highlighting its potential role in regulating cellular redox balance (60). GB treatment significantly inhibits lipid peroxidation and oxidative stress in both HFD-induced NAFLD mice and palmitic acid (PA)/oleic acid (OA)-treated HepG2 cells, with a pronounced effect on ferroptosis pathways. This protective effect is mediated through the activation of the Nrf2 pathway, which upregulates key antioxidant proteins, including GPX4, HO-1, TFR1, and FTH1, thereby mitigating ferroptosis (55).

Lip-1, a spiroquinoxaline derivative, exhibits potent and selective inhibitory effects on iron-dependent lipid peroxidation pathways, making it a promising candidate for targeting ferroptosis. Lip-1 exerts its effects by disrupting iron ion metabolism, mitigating lipid peroxidation-induced cellular damage, and demonstrating neuroprotective properties in cellular models (47). Given its ability to inhibit lipid peroxidation, Lip-1 has garnered significant interest for its potential therapeutic applications in metabolic diseases, including NAFLD and NASH. Lip-1 treatment alleviates hypertriglyceridemic pancreatitis by modulating lipid metabolism, inhibiting ferroptosis, and mitigating ER stress (61). In liver disease models linked to metabolic syndrome, Lip-1 reduces hepatic steatosis and mitochondrial ROS dysfunction, thereby slowing disease progression (52). Similarly, in NASH models, prophylactic Lip-1 supplementation significantly reduces hepatic steatosis and improves liver histology (38).

α-Tocopherol, also known as vitamin E, is a potent natural antioxidant that also functions as a ferroptosis inhibitor, effectively attenuating cellular lipid peroxidation and inhibiting ferroptosis (62). The anti-ferroptosis activity of α-tocopherol is mediated by Alox15, a key enzyme responsible for the generation of 4-hydroxy-2-nonenal (4-HNE), one of the final products of lipid peroxidation (63, 64). Previous studies have shown that 4-HNE protein adducts are significantly elevated in the livers of NAFLD patients, indicating elevated levels of lipid peroxidation (48). In a mouse model of MCD-induced liver injury, supplementation with α-tocopherol attenuated the MCD-induced increase in hepatic lipid peroxidation and restored the levels of GSH and SOD (49, 50). Recent research suggests that α-tocopherol supplementation reduces intracellular iron accumulation, attenuates lipid peroxidation, upregulates GPX4 expression, and restores mitochondrial membrane potential, thereby mitigating hepatotoxicity. This protective effect is mediated through the upregulation of Nrf2 expression (51). Similarly, oral supplementation of α-tocopherol in mice inhibits iron accumulation and modulates Nrf2 expression, leading to the depletion of hepatic iron stores and enhanced iron efflux through FPN, further strengthening the antioxidant response (65). However, the molecular mechanisms underlying the protective effects of α-tocopherol in ferroptosis-associated NAFLD remain poorly understood, offering an exciting direction for future research.

Despite their potent anti-ferroptotic effects, these inhibitors face significant challenges in clinical translation, primarily due to pharmacokinetic and physicochemical limitations. Fer-1 suffers from poor water solubility, which severely limits its bioavailability and hinders its therapeutic potential (66). These physicochemical properties impair its distribution and absorption, while also rendering it prone to oxidation and conferring a short half-life (67, 68). These pharmacokinetic challenges undermine its sustained efficacy, and the need for frequent dosing may elevate the risk of adverse effects, further limiting its clinical utility. Similarly, Tβ4 has a short biological half-life, requiring frequent dosing to maintain therapeutic levels, which diminishes its clinical practicality (69). Moreover, Tβ4 has a narrow therapeutic window, where both excessive and insufficient doses can result in suboptimal therapeutic outcomes or adverse effects, further complicating its clinical application (70).

Despite its potent antioxidant properties, the clinical utility of GB is hindered by poor bioavailability and inefficient delivery to target tissues, limiting its therapeutic efficacy (71). Lip-1 is rapidly metabolized and exhibits a short elimination half-life, which may compromise its therapeutic efficacy and reduce patient compliance due to the need for frequent dosing (21). Notably, most preclinical studies have relied on parenteral administration rather than oral formulations, indicating potential challenges in achieving adequate oral bioavailability and limiting its clinical applicability.

While high-dose α-tocopherol supplementation may exert therapeutic effects in specific contexts, the current evidence on the safety profile of high-dose α-tocopherol supplementation is still inconclusive. The recommended daily intake of 1,073 milligrams of RRR-α-tocopherol or its esters is currently recommended, yet the safety implications of exceeding this dosage warrant further investigation (72). Moreover, notable differences in bioavailability are observed between natural and synthetic α-tocopherol isoforms, with the natural form exhibiting superior biological activity. These limitations collectively hinder the clinical translation of ferroptosis inhibitors.

### 4.2 Intermittent fasting and time-restricted eating

Intermittent fasting (IF) and time-restricted feeding (TRF) are two widely studied dietary interventions, with IF involving strict caloric restriction within a limited time window, and TRF typically restricting caloric intake to specific hours of the day, commonly implemented as the 16:8 protocol. The protective effects of fasting have been extensively documented in a wide range of organisms, spanning from simple model organisms, such as yeast, nematodes, and fruit flies, to mammalian models, including mice and primates. These organisms have exhibited significantly extended lifespans under conditions of fasting or nutrient deprivation (73, 74). Fasting induces a metabolic shift from glucose utilization to fatty acid oxidation and ketone body production as primary energy sources. Additionally, ketone bodies act as potent signaling molecules, activating downstream protective pathways that modulate metabolic disorders, such as NAFLD and NASH. These pathways are mediated by factors like PPARγ coactivator 1α (PGC-1α) and fibroblast growth factor 21 (FGF21) (75). To explore the association between fasting and NASH, researchers established a NASH mouse model induced by a high-sugar, high-fat diet. Their results demonstrated that TRF significantly attenuated liver injury, decreased hepatic iron accumulation, and suppressed ferroptosis, thereby ameliorating NASH pathology. Moreover, ferroptosis was implicated in the pathogenesis of NASH, particularly through the inhibition of PPARα signaling. The study also identified a significant association between the circadian rhythm gene Per2 and ferroptosis, with hepatocyte-specific knockout of Per2 markedly suppressing hepatic ferroptosis (76).

## 5 Exercise regulates ferroptosis

Ferroptosis is a critical pathophysiological mechanism closely associated with the pathogenesis and progression of multiple diseases. Exercise, as a multifaceted health-promoting intervention, plays a pivotal role in the prevention and management of chronic diseases by modulating lipid metabolism, mitigating oxidative stress, and suppressing inflammatory responses (77). Furthermore, exercise can reduce an organism’s susceptibility to ferroptosis, providing novel insights into the prevention and therapeutic strategies for ferroptosis-related diseases (Table 3). Studies have demonstrated that moderate-intensity aerobic exercise can restore oxidative stress homeostasis by augmenting endogenous antioxidant defenses. This exercise-induced activation of various antioxidant pathways significantly decreases intracellular ROS levels, thereby inhibiting ferroptosis and ameliorating conditions such as osteoarthritis (78). Recent findings indicate that moderate-intensity exercise can suppress synovial ferroptosis through the upregulation of lipoxin A4 (LXA4) levels, thus alleviating knee osteoarthritis damage. This therapeutic effect is partially mediated by the activation of estrogen receptor β (ERβ) (79). Furthermore, exercise modulates bone health by promoting the release of irisin from bone cells. Under mechanical stimulation from exercise, increased irisin levels facilitate the removal of excess iron ions from bone cells, thereby preventing ferroptosis and preserving bone structural integrity. This discovery offers a novel theoretical basis for the prevention and management of osteoporosis (80). Moreover, studies have shown that ferroptosis is associated with cardiac dysfunction and mitochondrial structural abnormalities. However, exercise has been proven to enhance cardiac function by upregulating the activity of myocardial oxidative stress-related enzymes, thereby increasing the expression of two key ferroptosis biomarkers, GPX4 and PTGS2, and inhibiting myocardial ferroptosis (81, 82). This finding provides further evidence supporting the cardiovascular benefits of exercise. In cerebral ischemia/reperfusion injury, the expression of ferroptosis-related proteins is markedly downregulated. However, exercise can counteract this effect by upregulating ferroptosis-related protein expression, thereby inhibiting ferroptosis (83, 84). Notably, in some cases, such as when SLC7A11 is downregulated by Erastin, the neuroprotective effects of exercise may be partially attenuated (83). After traumatic brain injury, a decrease in GPX4 expression and an increase in lipid peroxidation are indicators of ferroptosis. However, these ferroptosis-related features are significantly mitigated by exercise intervention (85). Additionally, research has shown that 8 weeks of aerobic exercise can suppress lipid peroxidation through the Xc-/GPX4 axis in neurodegenerative diseases, thereby reducing neuronal sensitivity to ferroptosis and improving spatial cognition, learning, and memory in mice (86).

**TABLE 3:** The use of exercise in ferroptosis.

Modes of exercise	Disease	Mechanism of action and targets	References
Aerobic exercise	Osteoarthritis	Upregulate NRF2, GPX4, SLC7A11, and inhibit NF-κB.	(78)
Aerobic exercise	Knee osteoarthritis	Upregulate LXA4 and NRF2 expression	(79)
Aerobic exercise	Osteoporosis	Upregulate NRF2 and FPN expression	(80)
Aerobic exercise	High-fat induced myocardial injury	Enhance the expression of NRF2 and GPX4	(81)
Aerobic exercise	Azithromycin-induced myocardial injury	Increase the expression of GPX4 and PTGS2	(82)
Aerobic exercise	Ischemia/reperfusion	Enhance the expression of NRF2, SLC7A11, GPX4.	(83)
Aerobic exercise	Ischemic brain injury	Upregulate GPX4 and SLC7A11 expression, downregulate ACSL4 expression.	(84)
Aerobic exercise	Traumatic brain injury	Upregulate GPX4 expression, inhibit STING expression	(85)
Aerobic exercise	Alzheimer’s disease	Upregulate Xc- and GPX4 expression	(86)

NRF2, nuclear factor erythroid 2-related factor 2; GPX4, glutathione peroxidase 4; SLC7A11, solute carrier family 7 member 11; FPN, ferroportin; ACSL4, Acyl-CoA synthetase long-chain family member 4; NF-κB, nuclear factor kappa B; STING, stimulator of interferon genes; PTGS2, prostaglandin-endoperoxide synthase 2; LXA4, lipoxin A4.

**FIGURE 3:**
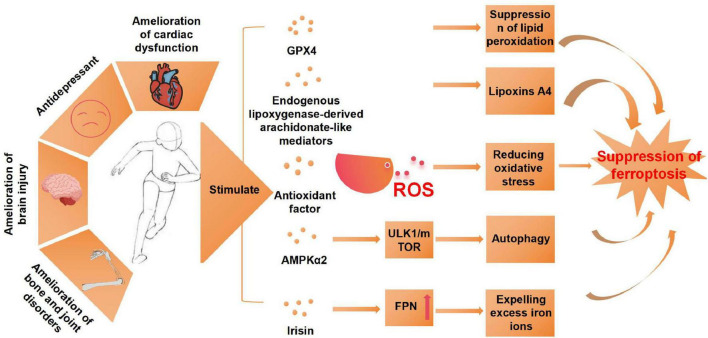
Exercise mitigates the effects of ferroptosis and its underlying mechanisms. AMPKα2, protein kinase AMP-activated catalytic subunit alpha 2; ULK1, unc-51-like kinase 1; mTOR, mammalian target of rapamycin; FPN, ferroportin; ROS, reactive oxygen species.

In summary, exercise modulates ferroptosis through multiple interconnected molecular pathways. The key mechanisms involve: (1) enhancement of antioxidant defense systems via the activation of NRF2, GPX4, and SLC7A11; (2) regulation of iron metabolism through FPN-mediated iron homeostasis; (3) suppression of lipid peroxidation via the ACSL4 and SLC7A11 pathways; (4) inhibition of inflammatory responses and cell death signaling through the NF-κB and STING pathways; and (5) improvement of mitochondrial function (Figure 3). These synergistic mechanisms collectively highlight exercise as a potent intervention against ferroptosis-induced cellular damage, thereby providing therapeutic potential for a range of ferroptosis-associated diseases. However, current research on the effects of exercise on ferroptosis is still limited, with the majority of studies concentrating on aerobic exercise interventions. Future studies should broaden their scope to investigate the impact of other exercise modalities on ferroptosis inhibition. Additionally, further research is required to elucidate the precise mechanisms underlying exercise-induced inhibition of ferroptosis, to comprehensively understand its biological underpinnings and clinical implications. The role of exercise in modulating ferroptosis is summarized in Table 3.

## 6 Conclusion

Ferroptosis, a recently discovered form of regulated cell death, is driven by iron-dependent lipid peroxidation and involves intricate molecular mechanisms. Emerging evidence has established a strong link between ferroptosis and the pathogenesis and progression of non-alcoholic fatty liver disease (NAFLD). This association is mediated through dysregulated iron metabolism, lipid peroxidation, and key signaling pathways, including the System Xc-/GSH/GPX4 axis, GCH1/BH4/DHFR pathway, and FSP1/CoQ10/NADH system. These findings highlight the potential of targeting ferroptosis as a novel therapeutic strategy for NAFLD, offering new avenues for disease management. This review also underscores the potential of exercise to inhibit ferroptosis, thereby modulating NAFLD progression and offering a non-pharmacological therapeutic option. Further research is essential to elucidate the dynamic interplay between exercise, ferroptosis, and NAFLD, providing a solid theoretical basis and practical guidelines for exercise-based interventions targeting ferroptosis inhibition. Despite promising findings, research on exercise interventions targeting ferroptosis in NAFLD is still in its infancy, with many unanswered questions and challenges. Key questions regarding optimal exercise modalities, intensities, and mechanisms of action remain to be addressed. While current studies have primarily focused on aerobic exercise, the effects of HIIT, resistance training, and combined exercise modalities on ferroptosis regulation are largely unexplored. Additionally, the differential effects of exercise intensity and training load on ferroptosis pathways remain poorly understood, representing a critical research gap. Nevertheless, exercise-based interventions targeting ferroptosis hold significant promise and may provide a novel, effective, and accessible therapeutic strategy for NAFLD patients.
